# An unusual outbreak of parvovirus B19 infections, France, 2023 to 2024

**DOI:** 10.2807/1560-7917.ES.2024.29.25.2400339

**Published:** 2024-06-20

**Authors:** Camille d’Humières, Anne Fouillet, Laura Verdurme, Stevens-Boris Lakoussan, Yves Gallien, Catherine Coignard, Marie Hervo, Anne Ebel, Anaïs Soares, Benoit Visseaux, Bruno Maire, Pierre-Henry Juan, Isabelle Parent du Châtelet, Jean-Paul Guthmann, Julien Durand

**Affiliations:** 1 Laboratoire Cerba, Frépillon, France; 2 Santé Publique France, Saint Maurice, France; 3 Laboratoire Eurofins Biomnis, Ivry sur Seine, France; 4 Laboratoire Eurofins Biomnis, Lyon, France; 5 Est-Rescue, Reims, France; 6 SOS Médecins association, Lyon, France; * These authors contributed equally to this work and share first authorship

**Keywords:** Parvovirus B19, outbreak, laboratory surveillance, syndromic surveillance, mortality, France

## Abstract

From April 2023 to May 2024, an unusual epidemic of parvovirus B19 (B19V) infections occurred in France. The number of B19V IgM-positive serologies was four times higher than in the previous epidemic in 2019. Clinical data from emergency networks corroborated this observation. Morbidity and mortality consequences were observed in children through all data sources. In adults, the increase was only observed in laboratory-confirmed data. Physicians and decisionmakers should be informed in order to better prevent, diagnose and manage at-risk patients.

Human parvovirus B19 (B19V) is a DNA virus responsible for a wide range of clinical symptoms from asymptomatic infections to very rare fatal events. Immunocompromised patients and patients with red blood cell disorders (e.g. sickle cell disease) are more likely to develop severe forms including death. Infection of pregnant women can cause in-utero death. Seasonal outbreaks of B19V are known to occur every 3–4 years in late winter and early spring [1].

Here we describe an ongoing and unusually large outbreak of B19V infections using laboratory-confirmed data from reference laboratories (3Labos network), clinical data from emergency departments (ED-OSCOUR network) and emergency general practitioners’ (GP) associations (SOS Médecins network (SOSM)), along with mortality data from death certificates in France.

## Human parvovirus B19 outbreak alert

In July 2023, the first alert concerning a major recrudescence of B19V infections in 37 children (of whom 21 had sickle cell disease) was sent by a paediatric hospital in the Paris region to the French public health agency Santé Publique France (SpFrance) [2].

**Figure 1:**
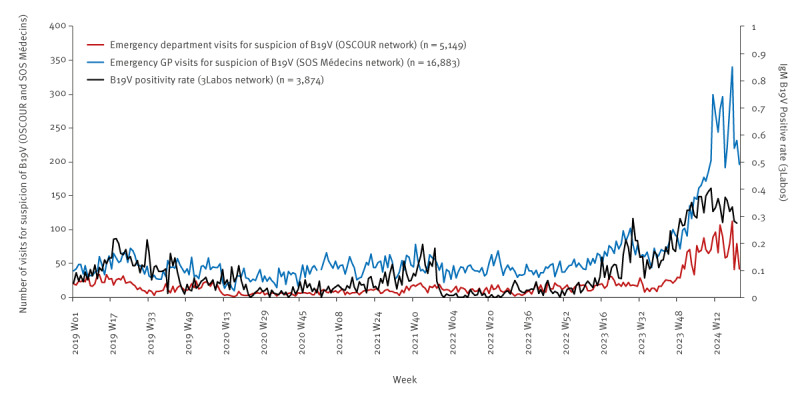
Weekly number of human parvovirus B19-related emergency consultations and B19V IgM positivity rate, children < 15 years, France, January 2019–May 2024

B19V: human parvovirus B19; ED-OSCOUR: network of emergency departments; GP: general practitioner; SOS Médecins: emergency general practitioner network; 3Labos: network of reference laboratories.

The investigations led by SpFrance confirmed a slight increase in the number of serologies positive for B19V IgM and in the number of B19V-related SOSM visits starting from April 2023 (Figure 1). After a short period of decline, a second increase was observed from November 2023 in laboratory-confirmed data and in the number of children visiting EDs and SOSM GPs. These indicators continued increasing until a peak in March 2024. We observed a decreasing trend in April and May 2024.

An increase in the number of deaths among infants and the number of positive PCRs performed on amniotic fluid was also reported up to March 2024. No deaths were reported in April and May 2024. 

## Exploring the outbreak with laboratory-confirmed data

The 3Labos network [3,4] supplies laboratory-confirmed data for a variety of health events to SpFrance for epidemiological surveillance purposes or in the context of emerging infectious threats and alerts [5]. The network is composed of Cerba (belonging to Cerba Healthcare) and Eurofins-Biomnis (Eurofins) laboratories, which perform specialised biological analyses on samples from community and hospital laboratories. The 3Labos network includes 5,969 sampling sites distributed across all regions of France (mainland and overseas); an overview of those sites is appended in the Supplement. The 3Labos network recorded 75% of the total B19V IgG and IgM serology reimbursements from the national health data system during the study period.

We analysed the number of serologies positive for B19V IgM and the B19V IgM positivity rates (PR) between January 2019 and the end of May 2024 in France. Results were grouped into three population categories: children younger than 15 years (who are at high risk of infection), women aged 20–40 years (since pregnant women are a high-risk population) and others.

**Figure 2:**
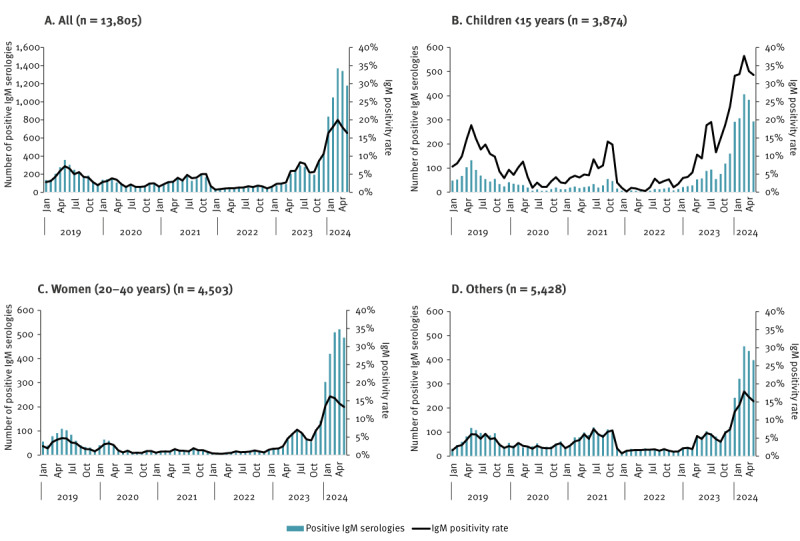
Number of positive parvovirus B19 IgM-positive serologies and positivity rate, by population category, France, January 2019–May 2024 (n = 13,805)

From 2019 to 2023, the yearly numbers of B19V IgM-positive serologies were 2,424, 1,102, 1,516, 533, 2,458 and from January to May 2024, it was 5,772. The previous epidemic had occurred in 2019 and reached a peak in May 2019 with 358 B19V IgM-positive serologies (7% PR) (Figure 2). After that, viral circulation remained very low during the COVID-19 pandemic years, especially in 2020 and 2022 (50–188 B19V IgM-positive serologies per month) in children and in 20–40-year-old women.

In 2023, we observed a first epidemic wave in late spring, with a peak in July (n = 296; 8% PR), corresponding to a threefold increase in the number of serologies positive for B19V IgM compared with March 2023 (before the beginning of the increase). After a short period of decline (−35% of B19V IgM-positive serologies between July and October), a second epidemic wave started in November 2023, reaching 1,370 B19V IgM-positive serologies (20% PR) in March 2024, a threefold increase compared with May 2019 (peak of the last epidemic wave). The seasonality was also unusual, as B19V outbreaks are usually restricted to late winter and early spring. All population categories were strongly affected, specifically women of childbearing age with 509 B19V IgM-positive serologies in March 2024. The positivity rate was particularly high among children, reaching 38% in March 2024. A decreasing trend was observed in April (33%) and May 2024 (32%).

The number of positive B19V PCRs and the PCR positivity rate showed the same trends (data not shown).

## Exploring the outbreak with clinical data

In 2004, SpFrance implemented the syndromic surveillance system SurSaUD based on two morbidity data sources: the ED-OSCOUR network and the SOSM network [6,7]. The system collects daily data from 700 EDs (96.6% of the national ED visits) and from 62 of 63 SOSM associations covering the whole national territory, including overseas. Data include demographic and administrative data, as well as medical diagnoses coded using international classification of diseases ICD-10 in EDs and a specific thesaurus for SOSM. ED visits related to B19V are identified with a medical diagnosis coded B08.3 or B97.6 in ICD10. Visits related to B19V within SOSM are coded with a generic code that also includes other infectious diseases (sepsis, Kawasaki disease).

**Figure 3:**
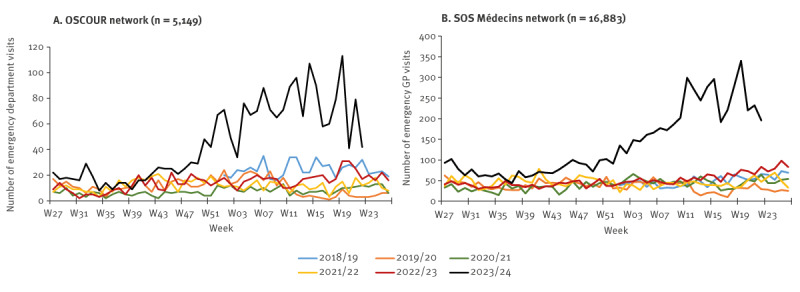
Weekly number of parvovirus B19-related visits to emergency departments and emergency GPs, children < 15 years, France, W01-2019–W22-2024

OSCOUR: network of emergency departments; GP: general practitioner; SOS Médecins: emergency general practitioner network.

From the beginning of 2019 to mid-2023, the weekly number of visits with suspicion of B19V infection to EDs and in the SOSM associations remained stable in children younger than 15 years (Figure 3). In July 2023, this number rose and slightly exceeded figures in the same period of the previous years, before returning to usual values between September and November 2023. 

From early December 2023, a major increase in the number of B19V-related visits in the two data sources was observed in children (Figure 3). The number rose from 29 ED visits in week 49 in 2023 (W49–2023) to 113 visits in W19–2024. The number of ED visits decreased slightly in W20-2023 and then remained stable (54 visits in average from W21 to W22-2024), above the numbers observed the previous years until the beginning of June. The increase was most prominent in children aged 6–10 and 3–5 years. The number of ED visits among people 15 years and older remained low and stable, including in women aged 20–40 years (data not shown). The number of B19V-related visits in EDs in children younger than 15 years (n = 1,768) remained above the expected number (n = 317, +458%) from 4 December 2023 to 2 June 2024.

The SOSM associations observed similar trends in the number of visits, with a major increase in children starting in W49–2023 (n = 72 visits) to a peak in W19–2024 (n = 340 visits) (Figure 3). From W49–2023 to W22–2024, 5,040 SOSM visits were recorded, compared with a mean of 1,148 visits during the same period of the four previous seasons (+339%).

## Severity: hospitalisations and B19V-related mortality

From 4 December 2023 to 31 May 2024 (W49–2023 to W22–2024), 1.8% (n = 32) of the 1,768 B19V-related ED visits in children younger than 15 years led to hospitalisation. This proportion was higher than the average proportion in the same period in the four previous seasons (1.2%).

The SurSaUD system also collects cause-related mortality data, which were extracted from both electronic and paper death certificates for the period 2019 to 2023 and from electronic death certificates for 2024 (43% of total mortality in France) [8]. We defined B19V-related deaths as those with a medical cause coded B08.3 in ICD10 or a B19V-related term when causes were available in free text.

On average, 1.8 B19V-related deaths were recorded per year during the period before the COVID-19 pandemic (2015–2019), with the majority (seven of nine) among people older than 15 years. From January to May 2024, based solely on electronic death certificates, five deaths were identified in children younger than 1 year. Four of these deaths occurred before the first 10 days of life and were linked to maternal–foetal B19V infection. No mention of comorbidity or immunosuppression was recorded in the other medical causes for the fifth death.

Based on data from Cerba laboratory, 16 of 224 (7.1%) PCRs performed on amniotic fluid had positive results for B19V from September 2023 to May 2024. The context of sampling was: nine intrauterine deaths, four hydrops foetalis, two intra-uterine growth retardations and one foetal anaemia. Only one positive PCR result had been recorded between 2019 and 2022.

## Discussion

Since April 2023, an unusual B19V outbreak has been ongoing in the French population, increasing until March 2024. The indicators showed decreasing trends in April and May, but remained above the values observed the previous years. The different indicators used to monitor the epidemic (IgM positivity rate, B19V IgM-positive serologies, B19V-related visits to SOSM GPs and EDs) all point towards this epidemiological trend in all metropolitan regions, in varying degrees of intensity (regional data not shown). While the number of deaths of children under 1 year of age (five deaths from January to May) in 2024 is based on non-exhaustive data, it highly exceeds the number between 0 and 1 in the previous 7 years (on exhaustive data). A recently published French study shows the same trend among B19V molecular screening in blood donors [9].

Key results were transmitted by SpFrance to the French ministry of health and sent to healthcare professionals through regional public health networks and learned societies. On 16 April 2024, public health authorities in France posted a message on EpiPulse European portal.

Other countries have also described a change in their B19V epidemiology. In particular, Israel experienced the largest and longest outbreak of B19V reported to date [10]. The epidemic is also observed in several European countries such as Denmark, Ireland, the Netherlands and Norway, as recently reported by the European Centre for Communicable Diseases Surveillance [11]. The impact of the B19 epidemic in pregnant women has recently been described in Denmark [12].

It has been suggested that the non-pharmaceutical interventions implemented during the COVID-19 pandemic were followed by an immunity gap that may have led to large outbreaks extending beyond the usual season and possibly affecting older children or presenting atypical clinical forms [13,14]. This has been shown for other pathogens such as *Streptococcus pyogenes* [15] or *Mycoplasma pneumonia* [16]. Several studies have shown a lower incidence of B19V infections during the COVID-19 pandemic [2,17]. While B19V infections are usually benign, patients with chronic anaemia, foetuses and immunocompromised patients are particularly vulnerable and risk life-threatening complications. During the current epidemic period, physicians and decisionmakers should be aware of the increasing cases of B19V infection in order to better prevent, diagnose and manage at-risk patients. 

This study underlines the value of epidemiological monitoring including complementary sources, to assess the extent and the severity of a health situation in the population. In this context, SpFrance maintains an enhanced surveillance based on these data sources to monitor the trend and possible public health impact of this epidemic and inform French health authorities [18].

## Conclusion

This work showed the unusual scale and seasonality of the current B19V epidemic in France, as well as its impact on morbidity and mortality. These data have direct and immediate use to inform health authorities and put in place recommendations and preventive measures. The use of existing non-specific surveillance systems and their adaptation to new health events is essential for effective epidemiological monitoring and control. 

## Supplementary Data

625000 Supplement
