# Supplementary material for: An unusual outbreak of parvovirus B19 infections, France, 2023 to 2024
Source: Euro Surveill. 2024 Jun 20;29(25):2400339. doi: 10.2807/1560-7917.ES.2024.29.25.2400339 (PMC11191415; doi:10.2807/1560-7917.ES.2024.29.25.2400339)

Supplement

This supplementary material is hosted by *Eurosurveillance* as supporting information alongside the article "An unusual outbreak of parvovirus B19 infections, France, 2023 to 2024", on behalf of the authors, who remain responsible for the accuracy and appropriateness of the content. The same standards for ethics, copyright, attributions and permissions as for the article apply. Supplements are not edited by *Eurosurveillance* and the journal is not responsible for the maintenance of any links or email addresses provided therein."

Number of sampling sites in 3Labos network by region, 2019-2024, France.

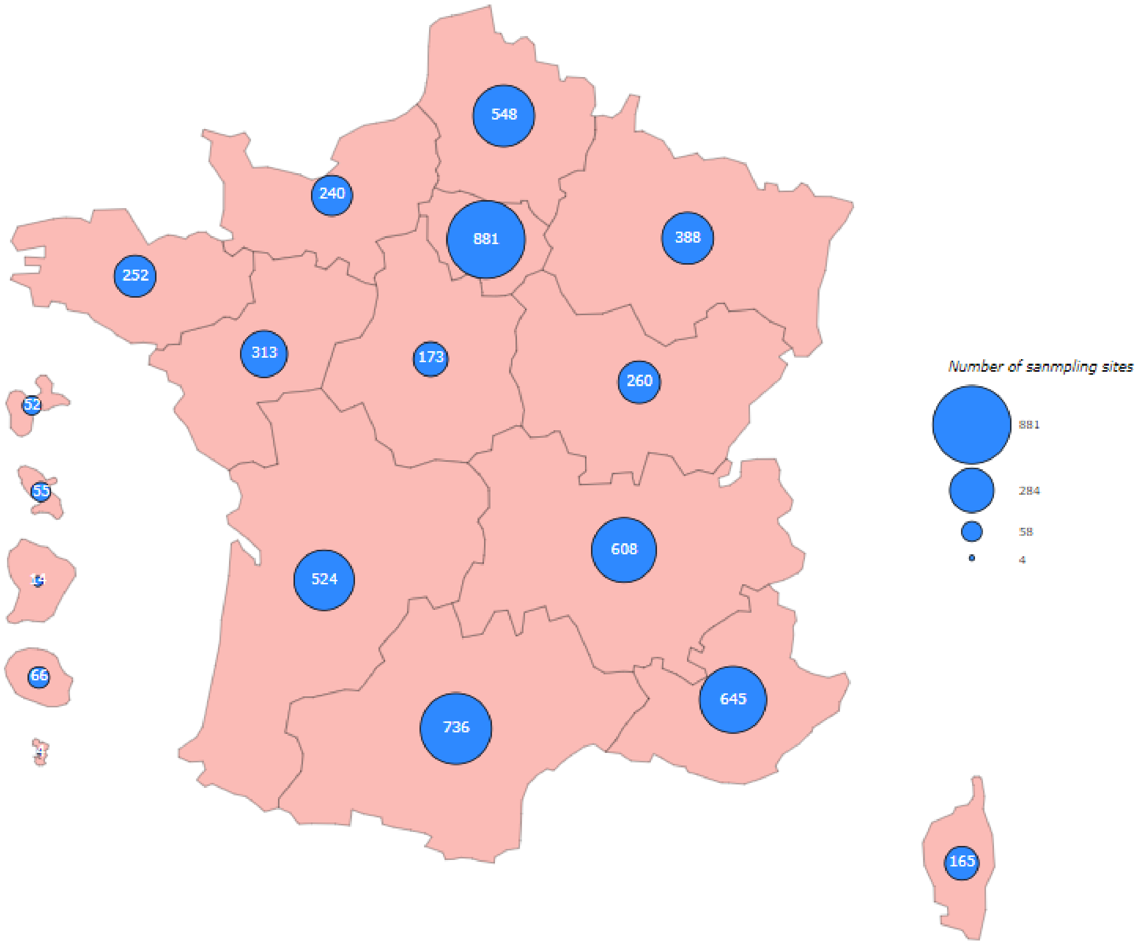

Supplement: Supplement [file 24-00339_FOUILLET_Supplement.pdf]
